# Clinical outcomes associated with the use of the NexSite hemodialysis catheter with new exit barrier technology: Results from a prospective, observational multi-center registry study

**DOI:** 10.1371/journal.pone.0223285

**Published:** 2019-10-07

**Authors:** Jeffrey G. Hoggard, Richard D. Blair, Manuel Montero, Moustafa A. Moustafa, Joseph Newman, Pablo E. Pergola, Nathan Saucier, Clarence J. Wheeler, Leonard A. Mermel, John R. Ross, Anatole D. Beserab

**Affiliations:** 1 Capital Nephrology Associates, Raleigh, North Carolina, United States of America; 2 Eastern Nephrology, New Bern, North Carolina, United States of America; 3 South Carolina Nephrology and Hypertension Center, Orangeburg, South Carolina, United States of America; 4 Eastern Nephrology, Greenville, North Carolina, United States of America; 5 Renal Associates PA, San Antonio, Texas, United States of America; 6 Kidney and Blood Pressure Clinic of Lubbock, Lubbock, Texas, United States of America; 7 Division of Infectious Diseases, Rhode Island Hospital and Department of Medicine, Warren Alpert Medical School of Brown University, Providence, Rhode Island, United States of America; 8 Access Connections LLC, Orangeburg, South Carolina, United States of America; 9 Division of Nephrology, Department of Medicine, Stanford University School of Medicine, Palo Alto, California, United States of America; University of Wisconsin, UNITED STATES

## Abstract

**Purpose:**

Decreasing the risk of catheter related bloodstream infections (CRBSIs) remains a key focus for improving outcomes and reducing cost of care for hemodialysis (HD) patients. Recent studies demonstrate CRBSI rates can be improved by managing bacterial colonization at the catheter exit site. Herein we present the results of a study documenting the clinical performance of the NexSite HD catheter, a new tunneled central venous catheter which incorporates Exit Site Management (ESM) technology.

**Methods:**

We conducted an observational study using a prospective, multi-center registry of HD patients implanted with the NexSite HD catheter. The primary endpoint for the study was CRBSI rate for a period up to 180-days following catheter placement. Secondary endpoints included device placement success rate, exit site healing, development of an exit site or tunnel infection, and early or late non-infectious catheter-related complications. All reasons for early non-elective catheter removal were recorded.

**Results:**

A total of 115 HD patients at 6 sites were included in the final analysis. Cumulative catheter use was 10,924 days with a mean duration of 95 days. Seven patients experienced CRBSIs during the study period resulting in a CRBSI rate of 0.64 per 1,000 catheter-days. Seventy-four patients (64.3%) had either elective catheter removal (n = 56) or utilized the catheter for the entire 180-day observation period (n = 18). Thirty-five patients (30%) underwent non-elective device removal either due to CRBSI (n = 5), low flow (n = 16), exit site issues (n = 7), or for other causes (n = 7). Six patients died during the observation period with 1 death due to CRBSI-associated complications and the remaining 5 deaths attributed to non-device related causes.

**Conclusion:**

Our findings demonstrate that the NexSite HD catheter equipped with ESM technology can achieve a CRBSI rate in compliance with the NKF KDOQI (National Kidney Foundation Kidney Disease Outcome Quality Initiatives) Clinical Performance Guidelines stated goal of less than 1.0/1,000 catheter-days when used in hemodialysis patients using current standard of care nursing protocols.

## Introduction

While the Fistula First Breakthrough Initiative has successfully increased the use of AV fistulas in the HD patient population [1], tunneled central venous catheters (CVCs) continue to play a vital role as a temporary “bridge” for patients who do not yet have a functioning AV access [2]. A lack of pre-dialysis vascular access planning, coupled with the time required for placement and successful maturation of AV fistulas, still results in an unwanted high prevalence and prolonged use of CVCs in this patient population [2, 3]. CVC use is associated with significant morbidity and mortality, contributing to poor patient outcomes and increased cost of care [3, 4]. This includes a two- to three-fold increase in risk of infection-related hospitalization or death compared to patients dialyzing via an AV access option [5].

The cost of diagnosing and treating CVC associated bloodstream infections on an outpatient basis, including the cost of blood cultures and antibiotics are included in the Centers for Medicare and Medicaid Services’ (CMS) End-Stage Renal Disease (ESRD) bundled payment to dialysis providers. Hospital admissions for catheter-related blood stream infections (CRBSIs) also result in decreased dialysis provider revenue resulting from missed dialysis sessions. From a payor perspective, the cost of hospitalization for CRBSIs has been reported to be from $17,000 to $32,000 per episode [6–9].

Reported CRBSI rates for tunneled CVCs in hemodialysis patients range from 1.1 to 5.5 per 1,000 catheter-days [5] with 30% to 60% of non-elective CVC removals being a result of bloodstream infections [10]. While the NKF-KDOQI Clinical Practice Guidelines for Vascular Access indicate that the adoption of best care practices is critical to achieving a CRBSI rate of 1.0 per 1,000 catheter-days [11], further reductions could be more consistently achieved using catheter technology that targets the sources of bacteria that contribute to CVC-related infections.

**Fig 1:**
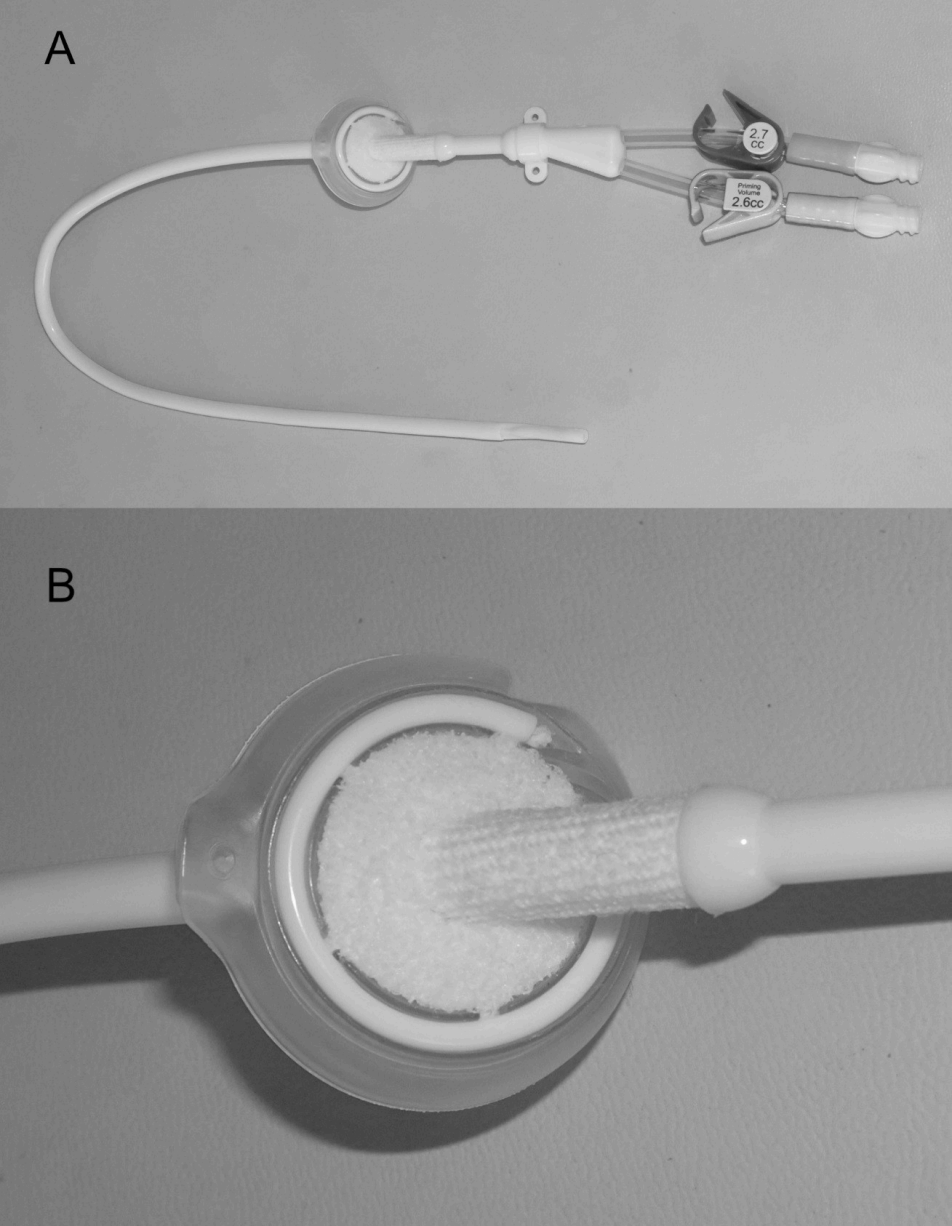
The NexSite HD Catheter showing A) the entire assembled catheter with B) a close-up view of the attached Dermal Ingrowth Support Collar (DISC).

CRBSIs occurs when microorganisms enter the bloodstream by one of two pathways; the catheter lumen or the catheter exit-site through the skin. While the lumen of a CVC is often thought to be the primary source of CRBSI events, there is strong clinical data demonstrating that the exit site can also be a significant contributing factor [12]. Recent studies suggest that effective catheter exit site management has the potential to significantly reduce CRBSI rates [12–14]. This hypothesis led to the development of the NexSite HD catheter (Marvao Medical Devices, Galway, Ireland), consisting of a CVC and a new Dermal Ingrowth Support Collar (DISC) component, both equipped with a Dacron tissue ingrowth cuff which are assembled at the exit site during placement (Fig 1). We report on a prospective, observational, multi-center registry study (NCT02453646) designed to document clinical results associated with the use of the NexSite HD catheter in ESRD patients who require a CVC for long term vascular access.

## Material and methods

The study was approved by New England Independent Review Board (IRB# 15–056). All patients signed written consents prior to enrollment and screened for conformance with the exclusion and inclusion criteria defined in the study protocol (S1 Table).

Exclusion criteria included any of the following: patients with a known bloodstream infection; treatment for a proven or suspected CRBSI within the 14 days prior to enrollment; fever (temperature ≥ 38.5°C) within 72 hours of planned catheter placement; antimicrobial therapy within one week of planned catheter placement; another indwelling catheter at the time of enrollment; or other conditions that the investigator believed should exclude the patient from the study. Patients previously implanted with a NexSite catheter were ineligible for reenrollment in the study. S1 Table lists the full inclusion and exclusion criteria associated with the study.

### The NexSite HD catheter

The NexSite HD catheter and DISC were packaged with accessories needed to facilitate tunneled CVC placement. The step-tipped version of the device which is FDA-cleared and CE marked was utilized for the present study device. Training on the placement and removal of the NexSite device was conducted at each site prior to initiating patient enrollment. Prophylactic antibiotics were given per each institution’s established protocols for catheter placement. The NexSite device was placed into a central vein of each patient with the distal tip of the catheter positioned in the right atrium. The catheter was tunneled from the exit site to the venotomy where it entered the selected vein, preferably the right internal jugular vein. The polyurethane catheter shaft is equipped with a Dacron cuff distal to the bifurcation hub. A separate polyurethane subcutaneous DISC (Dermal Ingrowth Support Collar), also equipped with a Dacron scaffold (Fig 1) is positioned subcutaneously at the exit site following the creation of a small skin pocket. The catheter cuff is aligned through the exit site and the DISC to promote tissue ingrowth into the Dacron scaffolds.

Following confirmation of correct tip positioning via fluoroscopy and determination of adequate blood flow through the catheter, the subcutaneous pocket and venotomy incisions are sutured closed, the catheter is secured to the skin, and the surgical site is cleaned and protected per the institution’s standard protocol. The NexSite catheter was then used to deliver hemodialysis therapy until permanent arteriovenous access was available or an adverse event necessitated catheter removal. Each dialysis center followed their own specific catheter maintenance and care procedures for the prevention of clotting and infection.

NexSite removal was achieved using blunt dissection around the catheter cuff to free it from the skin followed by gently pulling the catheter to remove it from the patient. The NexSite DISC incorporates a tab which enables it to be gripped with a hemostat and pulled to unravel the DISC allowing it to be easily removed through the exit without the need to make a pocket incision. The DISC’s Dacron cuff was then removed using a clamp.

Patient demographics, catheter insertion location, insertion date and removal date were collected for all patients. Charts for patients enrolled in the study were reviewed by study coordinators on a monthly basis until device removal or until 180 days after placement. The reason for elective or non-elective catheter removal was documented. Elective removal was typically due to the availability of a permanent arteriovenous access or other non-device related reasons which resulted in the patient no longer requiring the catheter. Non-elective removal was attributed to device-associated complications which included CRBSIs, exit-site issues, including infections or complications, or poor flow. All adverse events occurring during device placement were noted, as well as all adverse events observed thereafter. Results from blood cultures along with the use of antibiotics or thrombolytics to maintain proper device function were also documented as indicated.

All infections were managed according to physician discretion and local treatment protocols. The decision whether to remove the NexSite catheter or not in patients with infections was left to the judgement of the physician(s) managing the patient. The choice of either thrombolytic therapy or catheter removal for patients with thrombosis/poor flow was also not directed by the study protocol and left to the clinical judgement of the attending physician.

The primary endpoint for the study was CRBSI per 1,000 patient-days. Secondary endpoints included device placement success rate, exit site healing, development of an exit site or tunnel infection, early or late non-infectious catheter-related complications that necessitated non-elective removal of the device, and cumulative device survival.

The occurrence of a bloodstream infection (BSI), and probable source of the infection, were determined by the investigator based on clinical symptoms, blood culture results, co-morbidities, and other clinical factors, regardless of whether or not the catheter was removed. Blood cultures to confirm the presence of BSI were performed per dialysis provider protocol. At the conclusion of the study, all available relevant clinical data was provided to a Clinical Events Committee (CE) to adjudicate whether or not the BSI was catheter-related.

## Results

**Fig 2:**
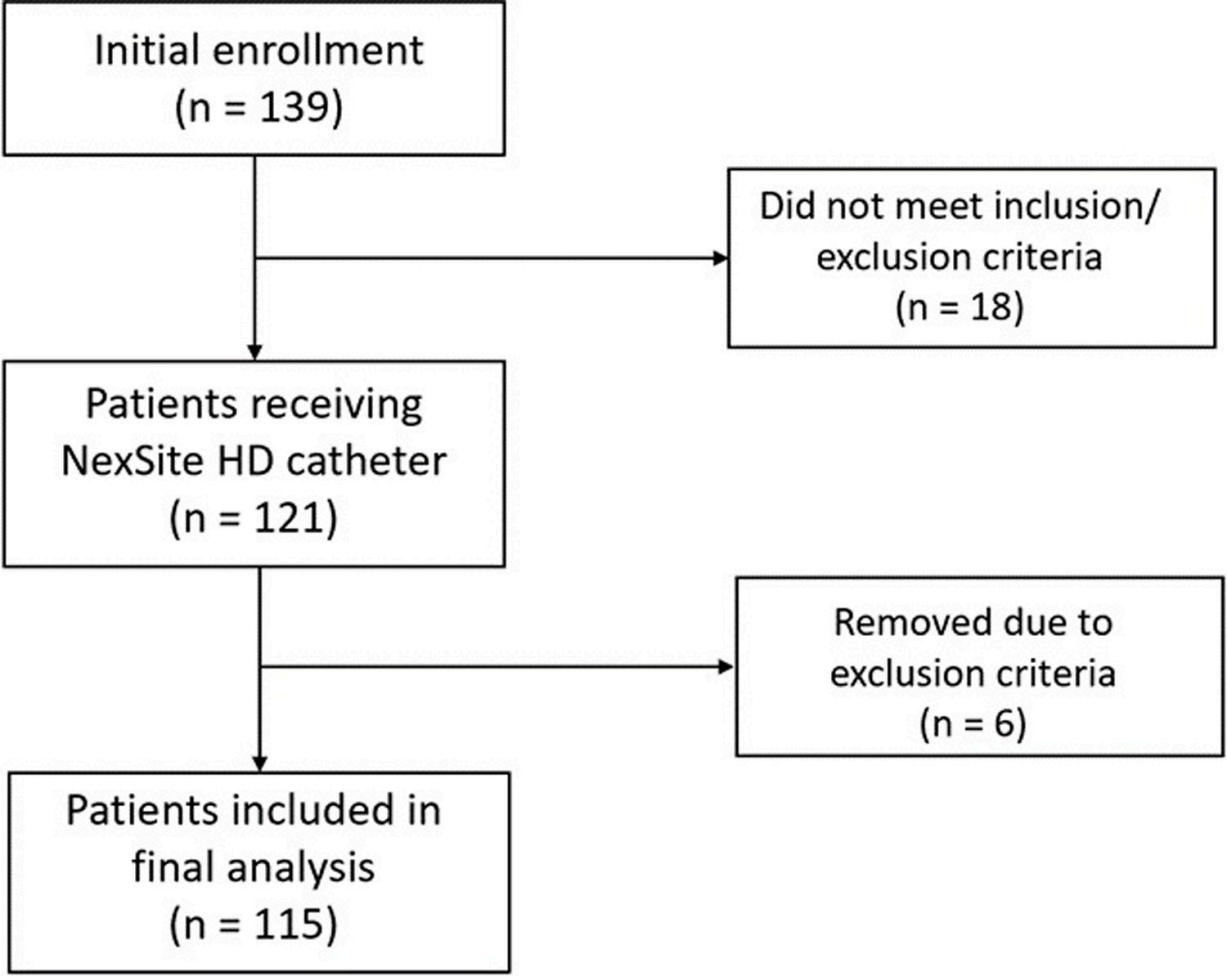
Disposition of patients enrolled in NextSite HD registry.

**Fig 3:**
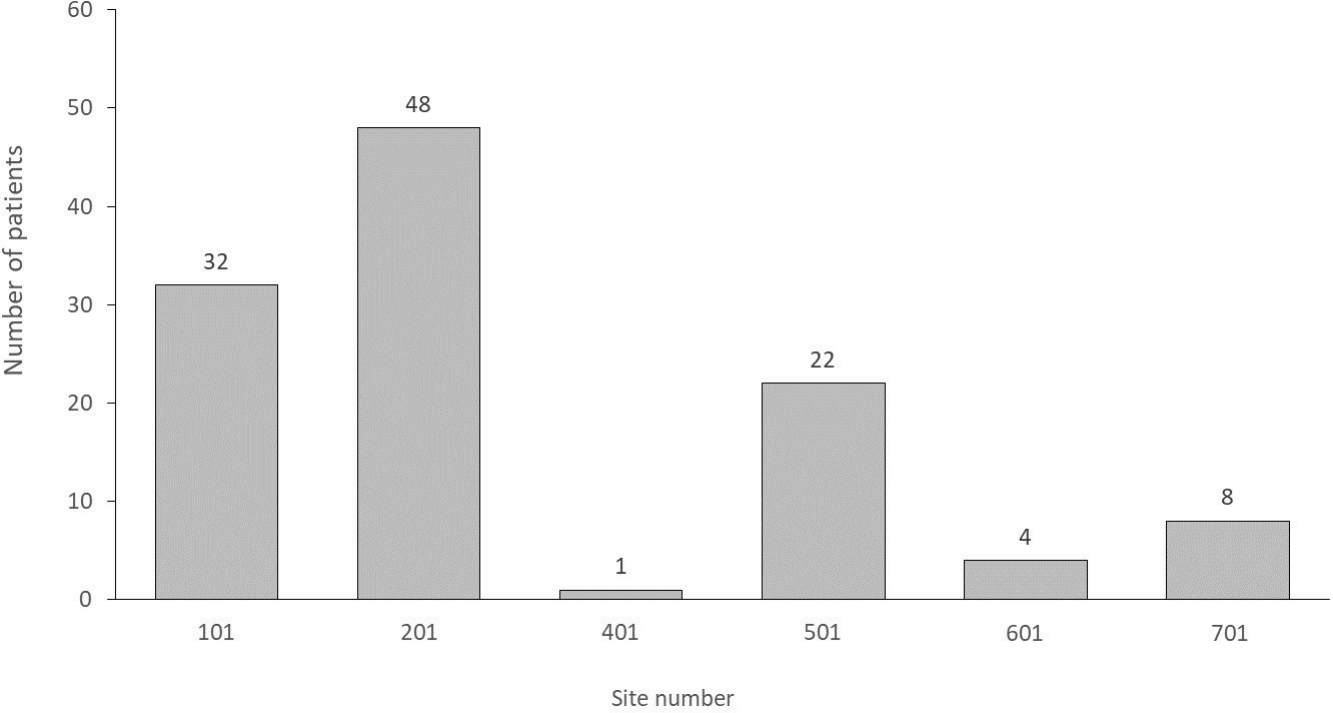
Patient enrollment by site.

A total of 139 HD patients at 6 sites were enrolled in the study with 115 patients included in the final analysis (Figs 2 and 3). Eighteen patients were excluded from the study prior to placement of the NexSite HD catheter for failing to achieve the inclusion criteria or for having one or more exclusion criteria. Another 6 patients were excluded following catheter placement as a result of having a study exclusion criterion which had not been identified during initial screening. This included two patients who were more than 80 years old, two patients with MRSA colonization, one patient with systemic lupus erythematosus (SLE), and one patient with a documented fever at the time of catheter placement.

### Patient demographics

Patients enrolled in the study had a mean patient age of 57 years and a mean BMI (Body Mass Index) of 31 (Table 1). Males represented 62% of the study population, 61% were diabetic, 53% were African-Americans.

**Table 1:** Patient demographics.

Mean age in years (range)	57 (20 to 80)
Gender	
Male (%)	71 (62)
Female (%)	44 (38)
Mean BMI (range)	31 (19 to 62)
Diabetic mellitus (%)	70 (61)
Ethnicity	
African-American	61 (53)
Caucasian	37 (32)
Hispanic	15 (13)
Native American	2 (1.7)
Venous Access Site (%)	
Right internal jugular	98 (85)
Right subclavian	1 (0.9)
Left internal jugular	15 (13)
Left subclavian	1 (0.9)

### Device placement

The NexSite HD catheter was successfully implanted in all 115 patients. No patients experienced a pneumothorax, hemothorax, arrhythmia, air embolism or arterial perforation during or following catheter placement. One patient experiencing tachycardia following the catheter placement procedure had their device removed on day 0. The number of catheters placed per site ranged from a single patient for site 401 to 48 for site 201 (Fig 3). Three sites who had experience with placement of the NexSite device prior to the study enrolled 55 patients into the registry and the remaining three sites which had no prior experience with the NextSite device enrolled 60 patients. The majority of catheters (85%) were placed in the right internal jugular vein (Table 1) with 13% placed in the left internal jugular vein. The remaining 2 patients (1.7%) had catheters placed in the right or left subclavian vein, respectively.

All catheter exit sites and pocket incisions healed within 2 to 4 weeks of device placement. Difficulty seating the catheter within the DISC during implantation occurred in one patient. This was resolved during the same procedure via the partial removal of the catheter and manipulation of the DISC in the pocket. There was no long term consequence associated with the above with catheter subsequently remaining in place for the entire 180 days of the study. One patient experience catheter dislodgement with associated bleeding from the exit site as a result of the device tugging on the patient’s shirt the day prior to planned catheter removal.

### Outcomes

**Fig 4:**
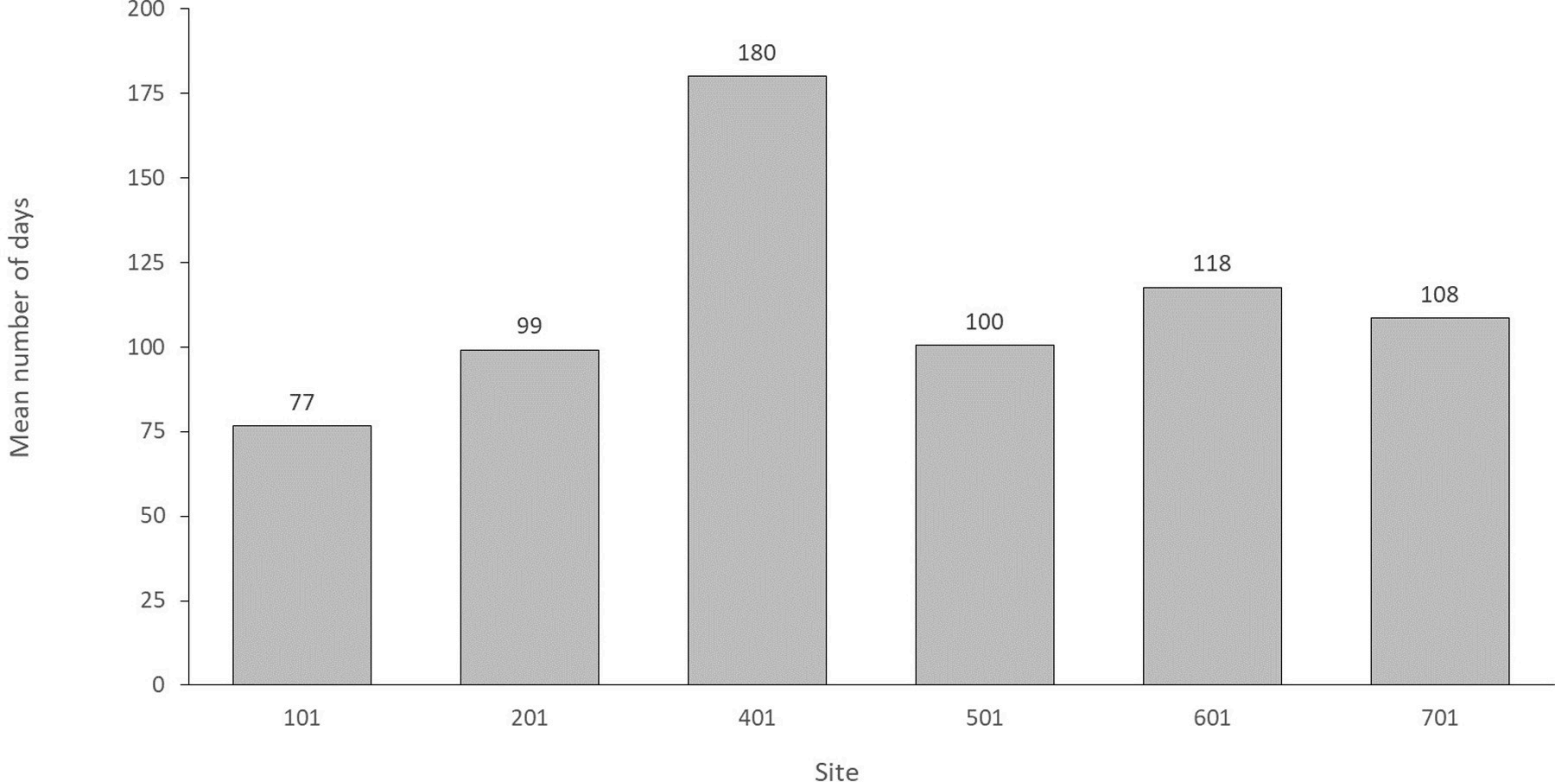
Mean duration of catheter use, per patient, by site.

Cumulative catheter use was 10,924 days (range 0 to 180 days) for the 115 patients included in the analysis with a mean per patient duration of catheter use of 95 days (Fig 4).

**Fig 5:**
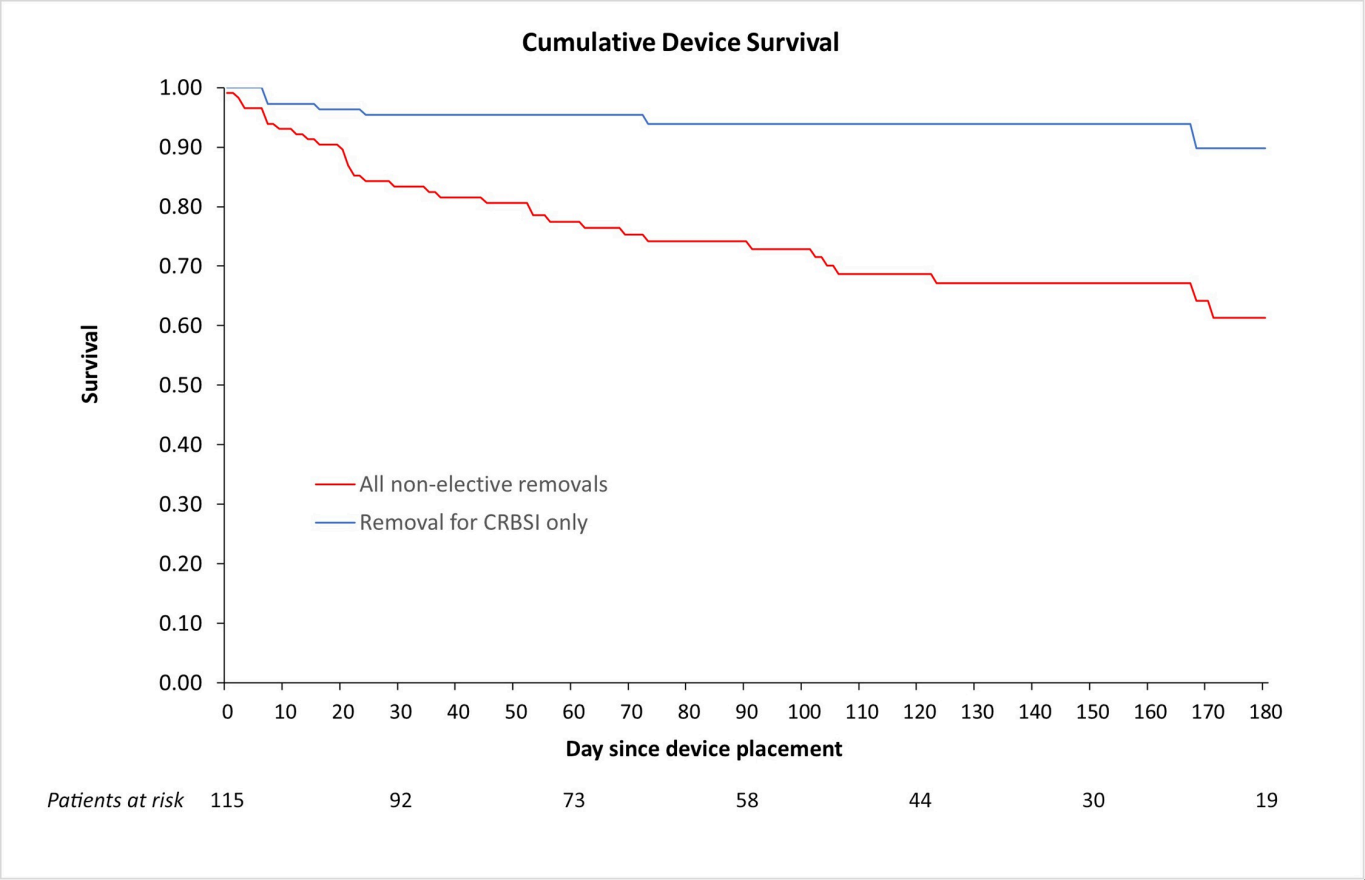
Kaplan-Meier survival curves for all non-elective device removals and removals for CRBSIs only.

Seven patients (6%) developed a CRBSI based on adjudication by the independent Clinical Events Committee, equating to a CRBSI rate of 0.64 events/1,000 catheter-days (Table 2). Fig 5 shows a Kaplan-Meier device survival curve for 5 patients who had non-elective device removals associated with CRBSI, 3 of which occurred in the first 30 days post placement. One patient developed a CRBSI which resolved following antibiotic therapy and did not require catheter removal. There was one CRBSI-associated death. No patients experienced a tunnel infection during the study. There were 10 confirmed exit site infections, equating to an exit site infection rate of 0.92 events/1,000 catheter-days. There was no correlation between the presence of an exit site infection and the development of a CRBSI.

**Table 2:** Reported culture results and associated outcomes for patients diagnosed with CRBSIs.

Patient	Organism	Outcome*
1	*Staphylococcus epidermis*	Resolved with antibiotics
2	*Staphylococcus aureus*	Non-elective device removal
3	*Enterococcus faecalis*	Patient death
4	*Staphylococcus aureus*	Non-elective device removal
5	Coagulase negative *Staphylococcus*, *Citrobacter freundii* and *Corynebacterium*	Non-elective device removal
6	Coagulase negative *Staphylococcus*	Non-elective device removal
7	*Staphylococcus aureus*	Non-elective device removal

*Decision to remove catheter was based on the clinical judgement of managing physician.

There were 22 incidents of catheter thrombosis/poor flow during the study period equating to a patency dysfunction rate of 2.01/1,000 catheter-days. In 6 of these cases, flow was restored using thrombolytics without the need for catheter exchange. There was one incident of superior vena cava syndrome which did not result in the need for catheter removal. No patients experienced catheter tip displacement.

There were 6 patient deaths over the course of the study, with 5 deaths attributed to non-device related causes after adjudication by the CEC. The remaining death was deemed to be CRBSI-associated. In this case, the patient presented to the emergency department with possible sepsis (nausea and malaise) approximately 4 months after device placement. Blood cultures grew *Enterococcus faecalis* and the patient died due to cardiac arrest. The CEC considered the death to be device-related because the blood cultures were positive and there was no other source of infection identified.

Seventy-four patients (64%) utilized the catheter until it was electively removed (n = 55) once it was no longer required, or for the entire 180-day observation period (n = 19). Thirty-five patients (30%) underwent non-elective device removal (Fig 5) due to low flow (n = 16), exit site issues, including both infectious and non-infectious causes (n = 7), CRBSI (n = 5) or for other causes (n = 7). CRBSIs were responsible for 14.3% of non-elective device removals. Detailed information for each non-elective device removal is shown in S2 Table.

## Discussion

Tunneled CVCs continue to play an important role in the delivery of hemodialysis therapy, primarily in patients awaiting placement or maturation of permanent AV access, but also for patients who either have exhausted all AV options for vascular access, or who are not otherwise suitable candidates for a surgically-placed AV access. The United States Renal Data System (USRDS) reports that 80% of patients initiating hemodialysis in the U.S. in 2015 utilized a CVC at their first outpatient hemodialysis session (3). Additionally, the USRDS reports that 15% of the prevalent U.S. hemodialysis patient population were still dialyzed with a CVC 12 months after initiating dialysis [3].

While implementation of the Fistula First Breakthrough Initiative has led to a significant increase in the placement and use of AV fistulas (1), 23% to 40% of AV fistulas created are never used for hemodialysis [15, 16]. Prolonged fistula maturation times and the sequential placement of secondary fistulas contribute to an increase in the time patients depend on a tunneled CVC as their primary means of vascular access.

Serious clinical complications associated with CVCs include infections, thrombosis, and central venous stenosis. CVC-related bloodstream infections occur at a rate of approximately 1.1 to 5.5 per 1,000 catheter-days (5). Catheter-related sepsis is also one of the most common causes of death in the HD population with studies demonstrating that patients dialyzed with a CVC have a higher mortality than those dialyzed using other AV access options [17]. The negative patient outcomes associated with CVC use result in increased in patient morbidity, and cost of care to payers and dialysis providers.

The present study was intended to document real world experience with the NexSite HD catheter. The results of our study demonstrate that the NexSite HD catheter is safe and effective alternative to conventional hemodialysis catheters. With NexSite, we achieved the KDOQI recommendation for the incidence of clinically significant insertion complications of 2% or less [11]. The adjudicated CRBSI rate of 0.64 per 1,000 patient days for the NexSite HD catheter is less than commonly reported CRBSI rates in the literature where standard catheter care protocols are used in a dialysis center setting, and it also achieved the KDOQI Clinical Performance Guideline goal of a CRBSI rate of less than 1.0 per 1,000 catheter-days [11].

Given that CVCs are a necessary option for many ESRD patients, the use of improved catheter designs can translate into better outcomes and lower costs. Recent improvements in catheter design, such as symmetrical tip catheter designs, have led to a reduction in catheter patency dysfunction but these advances have not led to reductions in CRBSI rates [18]. Antimicrobial catheter coating technologies have also so far failed to achieve documented reductions in CRBSI rates on tunneled CVCs for long-term use in the hemodialysis patient population [19]. The NexSite HD catheter provides a unique catheter-based approach to reducing the incidence of CRBSIs. The large surface of dermal tissue ingrowth scaffold material (Dacron) positioned at the exit site permits healing of the skin which establishes a natural barrier to entry of microorganisms along the external catheter surface of the indwelling length of the device.

The patency dysfunction rate of 2.0 per 1000 catheter-days we observed in the present study is consistent with step tip catheter designs such as the one used for the study. A newer version of the NexSite catheter that combines the ESM technology with a symmetrical tip catheter design may help to reduce patency dysfunction rates. Additional studies are needed to confirm this.

The need to create the subcutaneous pocket for the NexSite device and assemble the device added additional steps to the implant procedure compared to conventional tunneled CVCs. While some of the investigators in the present study had no prior experience with placing the NexSite device and limited, if any, experience creating subcutaneous pockets, the creation and closure of the subcutaneous pocket only added approximately 10 minutes to the placement procedure. Physicians who had no experience with device placement prior to the study reported that they were able to effectively create the subcutaneous pocket and implant the NexSite catheter after a brief training on the proper device placement technique in conjunction with a slide presentation describing the technique. As with all CVC placements, appropriate selection of the exit site and tunneling of the catheter are necessary to minimize the risk of catheter kinking and early low flow rate problems. Based on the results reported in our study, we believe that any physician experienced with catheter placement can rapidly gain proficiency with the placement of the NexSite catheter.

Device removal, including peeling of the DISC through the exit site after the catheter has been removed, was straightforward and able to be performed in an office setting. Locating and grasping the DISC tab to enable removal of the DISC was easily performed and became routine as more experience was gained.

Our study has several limitations. This includes the lack of a control arm and randomization of patients to be implanted with the NexSite device or a standard hemodialysis catheter. As a result, it is not possible to make a direct comparison of our results with those obtained with other catheters. Similarly, the adjudication committee was not blinded. We also did not collect cost data that would permit an analysis of the potential economic implications associated with the use of this catheter.

## Conclusion

In summary, NexSite HD catheter use was associated with a CRBSI rate of less than 1.0 per 1000 catheter-days in compliance with the K-DOQI clinical performance guidelines recommendation. The low CRBSI rate we observed suggests that the incorporation of a dermal ingrowth scaffold at the catheter exit site promotes the establishment of a natural barrier to the entry of microorganisms which contribute to the development of CRBSIs.

## Supporting information

S1 Table Inclusion and exclusion criteria. (DOCX)

S2 Table Detailed information on causes of non-elective device removals. (DOCX)
