# Supplementary material for: Clinical outcomes associated with the use of the NexSite hemodialysis catheter with new exit barrier technology: Results from a prospective, observational multi-center registry study
Source: PLoS One. 2019 Oct 7;14(10):e0223285. doi: 10.1371/journal.pone.0223285 (PMC6779244; doi:10.1371/journal.pone.0223285)
Supplement: S1 Table — (DOCX) [file pone.0223285.s001.docx]

**Supporting Information**

**S1 Table. Inclusion and Exclusion Criteria**

| *Inclusion Criteria:* |
| --- |
| - The patient shall be 18 - 80 years old. |
| - The patient requires the placement of a long term tunnelled HD catheter for administration of hemodialysis. |
| - The patient has anticipated survival in excess of 90 days from the anticipated date of catheter placement. |
| - The patient has the ability to understand the research subject information and sign a written Informed Consent form which must be obtained prior to initiation of the study. |
| - The patient receives the catheter via the internal jugular or subclavian vein. |
| - All female patients of child bearing potential must complete a negative pregnancy test and will confirm that they are not breastfeeding. |
| *Exclusion Criteria:* |
| - The patient is participating in another IRB (Institutional Review Board) clinical trial, which modifies standard-of-care treatment and/or involves an investigational device or drug. |
| - The patient has a confirmed or suspected infection, bacteremia or septicemia. |
| - The patient's physiology is NOT suitable for placement of the NexSite device; this will include an examination of the anatomy at the proposed catheter exit site. |
| - The patient is known or suspected to have allergies to the materials used in the construction of the device. |
| - The patient has previously suffered from coagulation issues or has had vascular surgery at the proposed placement site. |
| - The patient has received radiation treatment at the proposed catheter placement site. |
| - The patient has severe chronic obstructive lung disease. |
| - The patient is pyretic within 72 hours (temperature ≥38° C or 100.4°F) prior to placement and/or has received antimicrobial drugs within two weeks prior to catheterization. |
| - The patient is female of child bearing potential not using adequate contraception. |
| - The patient has another indwelling catheter. |
| - The patient has non-healing diabetic foot ulcers. |
| - The patient did not give informed consent. |
| - The patient would be unavailable for follow-up. |
| - The patient is a permanent nursing home resident. |
| - The proposed access site is not the internal jugular or subclavian vein. |
| - The patient is scheduled to undergo an elective surgical procedure (other than a procedure to create a graft or fistula) within the study timeframe. |
| - Any other condition that the Investigator believes should exclude the patient from the study. |
| - The patient does not have English or Spanish as their first language. |
