# Supplementary material for: Clinical outcomes associated with the use of the NexSite hemodialysis catheter with new exit barrier technology: Results from a prospective, observational multi-center registry study
Source: PLoS One. 2019 Oct 7;14(10):e0223285. doi: 10.1371/journal.pone.0223285 (PMC6779244; doi:10.1371/journal.pone.0223285)
Supplement: S2 Table — (DOCX) [file pone.0223285.s002.docx]

**Supporting Information**

**S2 Table. Detailed Information on Causes of Non-Elective Device Removals**

| **Patient ID #** | **Study Site** | **Catheter days** | **Reason for Device Removal** | **Comments** |
| --- | --- | --- | --- | --- |
| 101001 | 101 | 7 | Low Flow | Physician noted that left internal jugular vein catheter placements often have flow problems |
| 201003 | 201 | 12 | Low Flow | Reason for low flow was not determined |
| 201004 | 201 | 91 | Low Flow | Reason for low flow was not determined |
| 101004 | 101 | 102 | Low Flow | TPA could not restore flow through catheter |
| 201005 | 201 | 29 | Low Flow | Reason for low flow was not determined. Possibly due to placement too close to the clavicle |
| 201008 | 201 | 35 | Exit Site Infection | Physician reported that device was placed too close to a pre-existing exit site |
| 201009 | 201 | 62 | Skin erosion | Placement technique identified as a mitigating factor in exit site problem |
| 201014 | 201 | 3 | Low Flow | Placement technique identified as a mitigating factor in low flow problem |
| 201015 | 201 | 45 | Exit Site Infection | Suspected exit site infeetion due to drainage around the exit site |
| 201016 | 201 | 53 | Exit Site Infection | Wound culture showed *Staphyloccus aureus* infection |
| 101008 | 101 | 3 | Low Flow | TPA could not restore flow through catheter |
| 101014 | 101 | 2 | Low Flow | TPA was not used in attempt to restore flow through catheter |
| 201026 | 201 | 106 | Exit Site Infection | Wound culture showed *Pseudomonas aeruginosa* infection |
| 501001 | 501 | 123 | Low Flow | Reason for low flow was not determined. Possibly due to pocket being formed too superficially |
| 201037 | 201 | 69 | Exit Site Infection | Placement technique identified as a mitigating factor in exit site problem |
| 201038 | 201 | 53 | Exit Site Infection | Enterobacter cloacae cultured from site |
| 201039 | 201 | 37 | Removed as a precaution | Non-compliant patient was changing her dressings at home |
| 201049 | 201 | 22 | Single positive Blood culture | CEC reviewed and concluded result as a test contaminant. Non-CRBSI |
| 201050 | 201 | 73 | CRBSI | Reported positive blood cultures (*Staphylococcus*) |
| 201054 | 201 | 171 | Exit Site Complication | CEC reviewed concluded that event was a pocket incision infection |
| 201055 | 201 | 20 | Low Flow | CEC reviewed the event and concluded that the tip was malpositioned |
| 201063 | 201 | 7 | CRBSI | *Staphylococcus aureus* blood cultures confirmed CRBI |
| 501003 | 501 | 0 | Removed as a precaution | Physician reported that patient had a tachycardia episode on day of placement |
| 501005 | 501 | 56 | Low Flow | CEC reviewed and attributed flow problems to fibrin sheath formation |
| 501007 | 501 | 24 | CRBSI | Coagulase negative Staph blood cultures confirmed CRBI |
| 201065 | 201 | 168 | CRBSI | Multiple organisms cultured in blood drawn through catheter |
| 101024 | 101 | 14 | Dysfunctional Thrombosis | Physician noted that catheter tip was too high in SVC - poor tip placement |
| 701001 | 701 | 104 | Low flow | CEC reviewed and attributed flow problems to fibrin sheath formation |
| 701002 | 701 | 21 | Low Flow | Reason for low flow was not determined |
| 501010 | 501 | 7 | Low Flow | left internal jugular vein catheter placements often have flow problems |
| 501013 | 501 | 21 | Low Flow | Physician noted that catheter tip was too close to vessel wall |
| 601004 | 601 | 16 | CRBSI | Staph aureus blood cultures confirmed CRBI |
| 101031 | 101 | 21 | Low flow | Physician noted that subclavian vein catheter placements often have flow problems |
| 101035 | 101 | 9 | SVC syndrome | Patient had a prior history of SVC syndrome - switched to femoral access |
| 501024 | 501 | 22 | Exit site infection | SC placement in morbidly obese patient may have contributed to exit site infection |
